# Computer-Aided Diagnosis with Deep Learning Architecture: Applications to Breast Lesions in US Images and Pulmonary Nodules in CT Scans

**DOI:** 10.1038/srep24454

**Published:** 2016-04-15

**Authors:** Jie-Zhi Cheng, Dong Ni, Yi-Hong Chou, Jing Qin, Chui-Mei Tiu, Yeun-Chung Chang, Chiun-Sheng Huang, Dinggang Shen, Chung-Ming Chen

**Affiliations:** 1 National-Regional Key Technology Engineering Laboratory for Medical Ultrasound, School of Medicine, Shenzhen University, Shenzhen, Guangdong 518060, P.R. China; 2 Department of Radiology, Taipei Veterans General Hospital and National Yang Ming University, Taipei 112, Taiwan; 3 Department of Radiology and Medical Imaging, College of Medicine, National Taiwan University, Taipei 100, Taiwan; 4 Department of Surgery, College of Medicine, National Taiwan University, Taipei 100, Taiwan; 5 Department of Radiology and Biomedical Research Imaging Center, The University of North Carolina at Chapel Hill, Chapel Hill, NC 27599, USA; 6 Department of Brain and Cognitive Engineering, Korea University, Seoul 02841, Republic of Korea; 7 Institute of Biomedical Engineering, National Taiwan University, Taipei 100, Taiwan

## Abstract

This paper performs a comprehensive study on the deep-learning-based computer-aided diagnosis (CADx) for the differential diagnosis of benign and malignant nodules/lesions by avoiding the potential errors caused by inaccurate image processing results (e.g., boundary segmentation), as well as the classification bias resulting from a less robust feature set, as involved in most conventional CADx algorithms. Specifically, the stacked denoising auto-encoder (SDAE) is exploited on the two CADx applications for the differentiation of breast ultrasound lesions and lung CT nodules. The SDAE architecture is well equipped with the automatic feature exploration mechanism and noise tolerance advantage, and hence may be suitable to deal with the intrinsically noisy property of medical image data from various imaging modalities. To show the outperformance of SDAE-based CADx over the conventional scheme, two latest conventional CADx algorithms are implemented for comparison. 10 times of 10-fold cross-validations are conducted to illustrate the efficacy of the SDAE-based CADx algorithm. The experimental results show the significant performance boost by the SDAE-based CADx algorithm over the two conventional methods, suggesting that deep learning techniques can potentially change the design paradigm of the CADx systems without the need of explicit design and selection of problem-oriented features.

Computer-aided diagnosis (CADx) is a computerized procedure to provide a second objective opinion for the assistance of medical image interpretation and diagnosis^1^,^2^,^3^,^4^,^5^,^6^,^7^,^8^,^9^,^10^. One of the major CADx applications is the differentiation of malignancy/benignancy for tumors/lesions^3^,^4^,^11^,^12^,^13^,^14^,^15^. Several studies have suggested that the incorporation of the CADx system into the diagnostic process can improve the performance of image diagnosis by decreasing inter-observer variation^16^,^17^ and providing the quantitative support for the clinical decision like biopsy recommendations^5^, etc. Specifically, the CADx systems were shown to be effective to assist the diagnostic workup for the reduction of unnecessary false-positive biopsies^6^ and thoracotomy^10^.

To achieve malignancy identification, the conventional design of CADx is often composed of three main steps: feature extraction^4^,^6^,^7^,^12^,^13^,^14^,^15^,^18^, feature selection^18^,^19^,^20^,^21^, and classification. These three steps need to be well-addressed separately and then integrated together for the overall CADx performance tuning. In most previous works, engineering on effective feature extraction step for each specific problem was regarded as the one of the most important issues^4^,^6^,^7^,^12^,^13^,^14^,^15^,^18^. Extraction of discriminative features could potentially ease the latter steps of feature selection and classification. Nevertheless, the engineering of effective features is problem-oriented and still needs assistance from the latter steps of feature selection and feature integration by classifier, to achieve accurate lesion/nodule differentiation. In general, the diagnostic image features can be categorized into morphological and textural features.

The extraction of effective features is *per se* a complicated task that requires a series of image processing steps. These image processing steps involve **1)** image segmentation^4^,^6^,^14^,^15^,^22^,^23^,^24^ for the morphological feature computing^4^,^6^,^7^,^14^,^15^,^22^, which unfortunately remains quite difficult to address^25^, and **2)** image decomposition^12^,^19^ followed with statistical summarizations and presentations^12^,^19^,^20^,^26^ for the textural feature calculation^27^. Accordingly, the extraction of useful features highly depends on the quality of each intermediate result in the image processing steps^4^,^6^,^14^,^22^,^27^, which often needs many passes of trial-and-error design to find satisfactory intermediate results. Meanwhile, the computerized image segmentation results often require various case-by-case user interventions, e.g., parameter adjustment^6^,^28^, manual refinement^6^,^9^,^28^,^29^,^30^,^31^, solution selection^4^,^23^, etc., to improve the contour correctness^4^,^6^,^13^,^15^,^23^. However, with the user’s intervention on the segmentation results, the differential diagnostic outcome from the CADx system will be biased and the objectiveness will no longer hold. In summary, the design and tuning of the overall performance of the conventional CADx framework tends to be very arduous, as many image processing issues need to be well resolved.

## Deep learning for CADx

Recently, the deep learning techniques have been introduced to the medical image analysis domain with promising results on various applications, like the computerized prognosis for Alzheimer’s disease and mild cognitive impairment^32^, organ segmentations^33^ and detection^34^, ultrasound standard plane selection^35^, etc., on 3D or 4D image data, etc. In the context of CAD, most works focused on the problem of abnormality detection (CADe)^36^,^37^,^38^. For the problem of CADx, a specific convolutional neural network model, OverFeat^39^, was employed in the work^40^ to classify the specific type of peri-fissural nodules with the ensemble fashion in AUC performance around 0.86. In this study, we further exploit the deep learning model of the stacked denoising autoencoder (SDAE)^41^ for the differentiation of distinctive types of lesions and nodules depicted with different imaging modalities.

The deep learning techniques could potentially change the design paradigm of the CADx framework for several advantages over the old conventional frameworks. The advantages can be three-fold. *First*, deep learning can directly uncover features from the training data, and hence the effort of explicit elaboration on feature extraction can be significantly alleviated. The neuron-crafted features may compensate and even surpass the discriminative power of the conventional feature extraction methods. *Second*, feature interaction and hierarchy can be exploited jointly within the intrinsic deep architecture of a neural network. Consequently, the feature selection process will be significantly simplified. *Third*, the three steps of feature extraction, selection and supervised classification can be realized within the optimization of the same deep architecture. With such a design, the performance can be tuned more easily in a systematic fashion.

**Figure 1:**
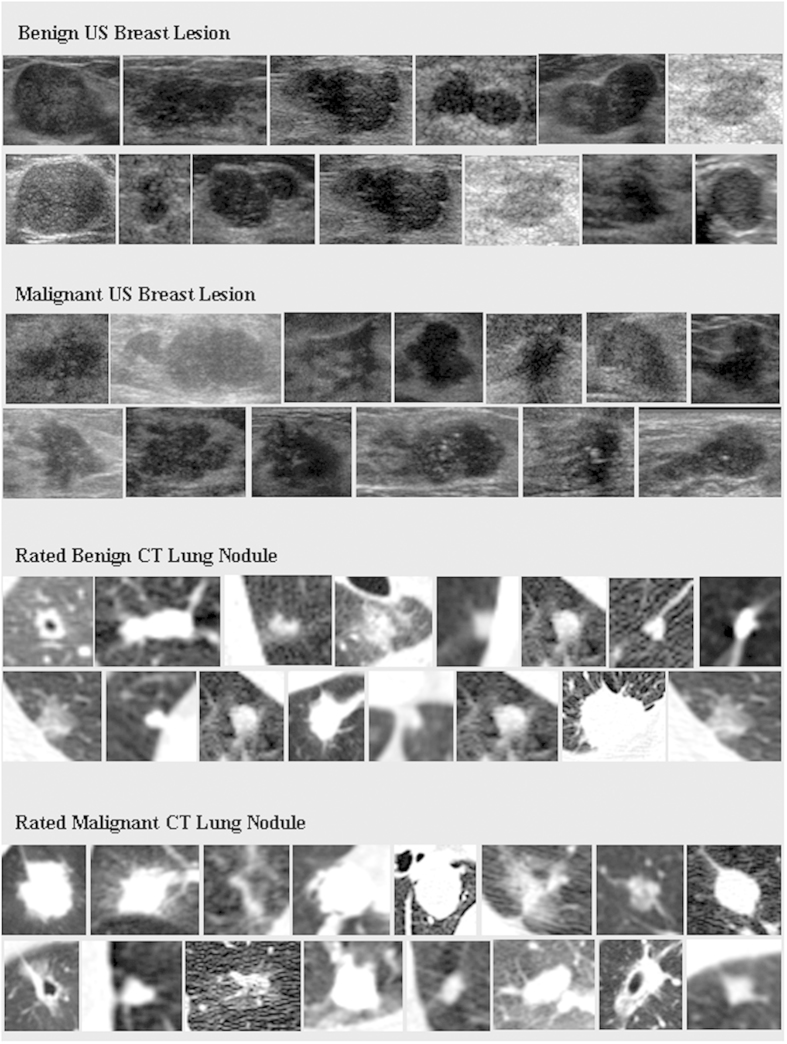
Exhibition of breast lesions and lung nodules in US and CT images.

The SDAE is a denosing version of the stacked autoencoder (SAE)^42^. The SAE/SDAE architecture can automatically discover the diverse representative patterns from the data with the intrinsic data reconstruction mechanism. Accordingly, the SAE/SDAE architecture can potentially address the issues of high variation in either shape or appearance of lesions/tumors. Meanwhile, with the advantages of automatic feature extraction mechanism and noise tolerance, the SDAE-based CADx scheme can circumvent the potential inaccurate image processing results that could lead to unreliable features in the conventional CADx framework. To show the wide scope of applicability, the SDAE-based CADx model is applied to the differential diagnosis of breast lesions in ultrasound (US) images and pulmonary nodules in CT images. Two CADx methods, i.e., RANK^19^ and CURVE^12^ that were specifically developed for the breast and lung applications, respectively, and clinical morphological features (MORPH) are implemented for performance comparison. The MORPH features are computed from either experts’ drawings or the computer-generated boundaries by level set^43^ (DRLSE) and grow-cut^44^ (GC) methods. Meanwhile, the effect of feature combination of RANK and CURVE with the MORPH is also explored. The experimental results show that the deep-learning-based CADx can achieve better differentiation performance than the comparison methods across different modalities and diseases. To illustrate the challenges of the two CADx applications, Fig. 1 lists several cases of breast lesions and pulmonary nodules depicted in US and CT images, respectively. The cases demonstrated in Fig. 1 may not be easy to differentiate for a person without a medical background and even for a junior medical doctor.

## Experiment Results

### Experiment setup

We compare the performances of the SDAE-based CADx and the two compared algorithms with the six assessment metrics: **1)** area under receiver operating characteristic curve (AUC), **2)** accuracy (ACC), **3)** sensitivity (SENS), **4)** specificity (SPEC), **5)** positive predictive value (PPV), and **6)** negative predictive value (NPV). For the robustness to data dependence, we perform 10 times of 10-fold cross validations for the SDAE and other baseline algorithms on both the breast US and lung CT datasets. In each cross validation, the partition of training and testing data is randomly determined. For the illustration of the performance dependency of the conventional CADx frameworks on the specific problems, all baseline algorithms are applied to all datasets.

Two slice selection strategies, i.e., SINGLE and ALL, are implemented for the lung CT CADx problem. For the SINGLE strategy, there are totally 1400 training and testing ROI samples. Each ROI sample stands for a distinctive nodule. In the ALL strategy, member slices of each nodule are involved in the training and testing. Specifically, the member slices of all 1360 training nodules in the 10-fold cross validation scheme are randomly permutated as the training samples. For the 140 testing nodules, the final differentiation of a testing nodule is determined with the majority voting from the member slices. In the ALL strategy, there are 10133 involved slices. Because slice thickness variation is very high (0.6–5 mm), fully 3D image features for the lung CADx may not be suitable.

The distinctiveness of each set of the 10-fold partition is assured in the random selection process. In each 10-fold cross validation, all algorithms share the same sample partition setting on each fold for fair comparison. For both lung and breast cases, we use the same SDAE architecture with 2 hidden layers, each containing 200 and 100 neurons, respectively. The true positive of the lung CT and breast US data is defined as the malignant class.

## Results

**Figure 2:**
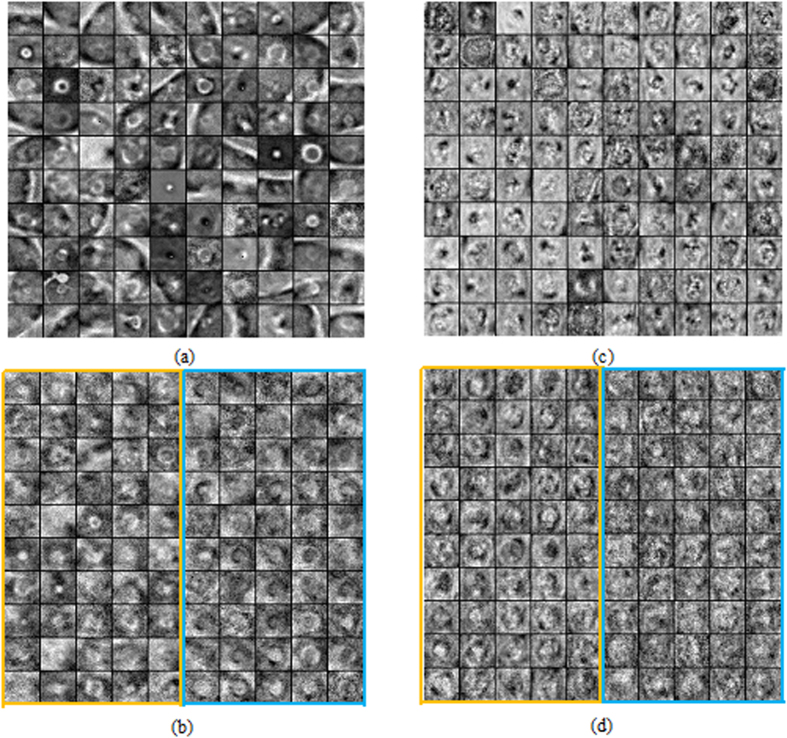
Examples of constructed patterns in the first and second hidden layers at the pre-training step: (**a,b**) patterns of the first and second hidden layers for pulmonary nodules; (**c,d**) patterns of the first and second hidden layers for breast lesions. SDAE architecture with two hidden layers is used in this study for the differentiation of pulmonary nodules and breast lesions. It is worth noting that the patterns of the second hidden layers are constructed as the weighted sums from all patterns in the first layer. In the reconstruction, the first layer neurons are simply all assumed activated. The neuron activation can be more complicated with the feed-in of real image data. In (**b**,**d**) the example patterns enclosed by the yellow rectangles hold the positive weightings to the RN nodule and benignant lesion classes in the supervised training step, whereas the patterns in blue regions are connected to the RM nodule and malignant lesion classes with positive weightings. It can be observed from (**b,d**) that the second hidden layer patterns appear fuzzier due to the effect of weighted sum. All patterns are normalized for clearer presentation.

To illustrate the patterns automatically discovered by SDAE, Fig. 2 lists several patterns of pulmonary nodules in CT images and breast lesions in US images, respectively, at the pre-training step. In the subfigures (a) and (c), the diverse appearance patterns of various nodules/lesions summarized at the first hidden layer are shown. The patterns at the second hidden layers that automatically encode the intertwined and hierarchical relations to the first hidden layer patterns are demonstrated in the subfigures (b) and (d).

**Figure 3:**
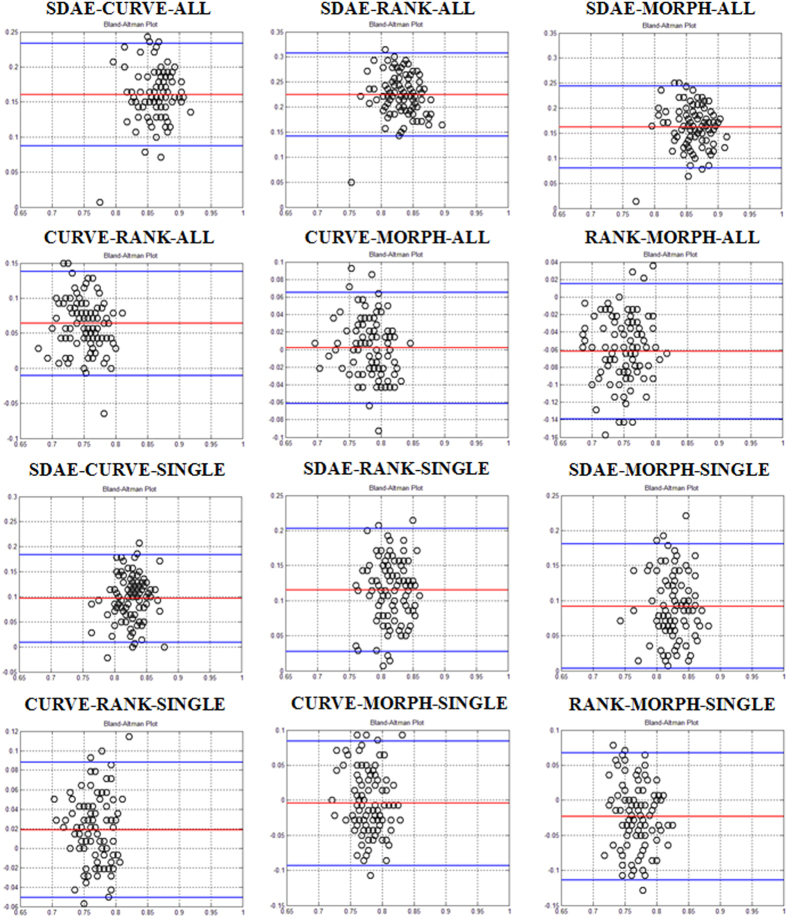
ACC Bland and Altman plots for six algorithm comparing pairs of “SDAE-CURVE”, “SDAE-RANK” , “SDAE-MORPH”, “CURVE-RANK”, “CURVE-MORPH”, and “RANK-MORPH” on the lung CT dataset. The comparing pairs with ending tag “ALL” are the results with the strategy of using all member slices of a nodule for the training and testing of the three algorithms. The pairs with tag “SINGLE” compare the computerized results with the slice selection strategy of using middle slice.

**Figure 4:**
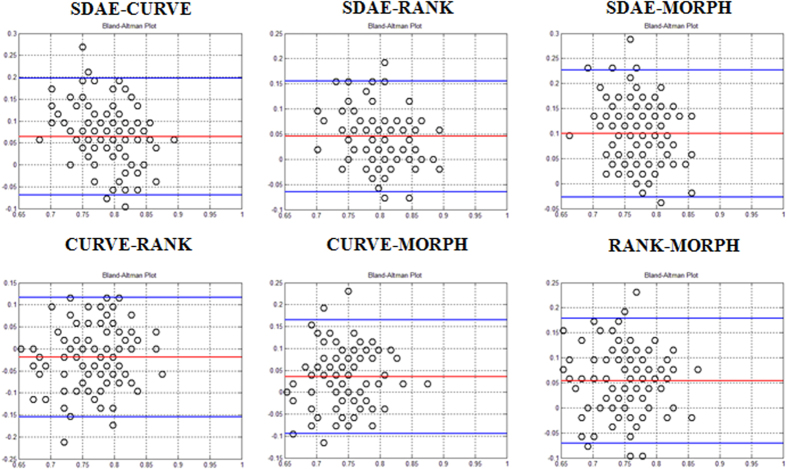
ACC Bland and Altman plots for performance comparison of the pairs “SDAE-CURVE”, “SDAE-RANK”, “SDAE-MORPH”, “CURVE-RANK”, “CURVE-MORPH”, and “RANK-MORPH” on the breast dataset.

Tables 1 and 2 summarize the statistics of six performance metrics for all algorithms on the lung CT and breast US datasets, respectively. To visualize the performance agreement between each pair of the comparing algorithms (SDAE, CURVE, RANK, and MORPH) over all 100 folds, Bland and Altman plots^45^ of the ACC metrics on the lung and breast datasets are drawn in Figs 3 and 4, respectively. In a Bland and Altman plot, the horizontal axis stands for the mean value of the assessment metrics between the two comparing methods, whereas the difference values of the two methods are coordinated in the vertical axis. The red line in the Bland and Altman plot suggests the mean difference of the assessment values, whereas the top and bottom blue lines delimit the ±1.96 standard deviation lines of the difference values.

**Figure 5:**
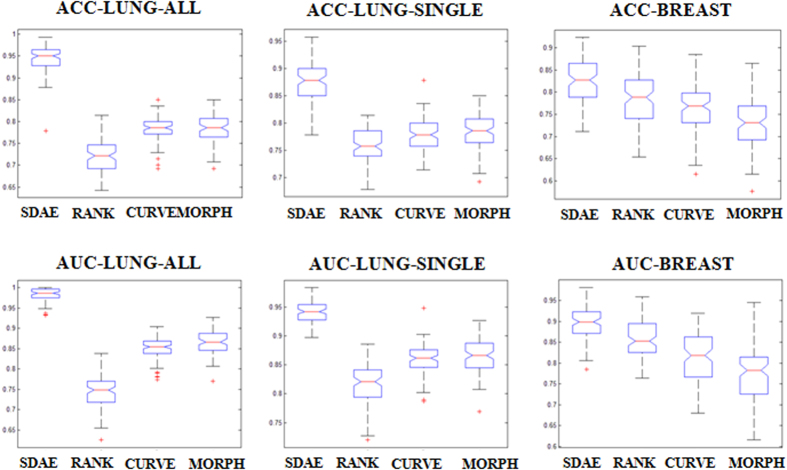
Box plots for performance for the lung and breast datasets with respect to the ACC and AUC metrics.

As can be observed from Figs 3 and 4 and Tables 1 and 2, the SDAE algorithm performs better than the other three algorithms. For the lung nodule differentiation, the SDAE algorithm at least achieves 0.15 and 0.1 higher ACC values than the CURVE, RANK and MORPH methods with respect to the ALL and SINGLE strategies in average. The CURVE algorithm achieves better than the RANK algorithm with the mean ACC differences for the ALL and SINGLE strategies, respectively. The mean ACC differences between the CURVE and MORPH are near to 0 with both strategies. The mean ACC differences between the RANK and MORPH are all lower than 0, suggesting that the RANK is averagely not able to outperform MORPH. For the breast application, the SDAE can achieve at least higher ACC values 0.04 than the RANK, CURVE, and MORPH in average. The CURVE perform worse than RANK with the mean differences of the ACC values around −0.02, but better than the MORPH with mean differences w.r.t. ACC values around 0.05. The RANK on the other hand can perform the classification of breast lesions better than using the clinical MORPH features with mean differences more than 0.05 on the ACC metrics. To further illustrate the ACC and AUC performance distributions of SDAE, CURVE, RANK and MORPH, the corresponding box-plots on the lung and breast datasets are also shown in Fig. 5. Meanwhile, it can be found from Tables 1 and 2 that the morphological features from the DRLSE and GC methods are not very helpful for the boosting of performance. It is because that satisfactory lesion/nodule boundaries are quite difficult to obtain without user intervention. The discussion about the image segmentation can be found in the second section of the supplementary.

Referring to the Fig. 5, it can be found that performance distributions appear substantially different in most cases, except the pairs “CURVE-MORPH-ALL” and “CURVE-MORPH-SINGLE”. To further investigate the significance of differences for these two pairs, the two sample t-test is applied. The ACC *p*-values of the pairs “CURVE-MORPH-ALL” and “CURVE-MORPH-SINGLE” are 0.69 and 0.32 respectively, whereas the AUC *p*-values w.r.t. each pair are 0.0001 and 0.088. It is thus suggested that ACC performance differences are not significant between the methods CURVE and MORPH on both strategy and the AUC performance between CURVE and MORPH is not significantly different on the SINGLE strategy.

## Discussion and Conclusion Remarks

The experimental results show that the SDAE algorithm outperforms the conventional CADx algorithms on both applications. Specifically, as can be observed in Table 1, Figs 3 and 5, the SDAE algorithm can differentiate lung nodules significantly better than the two baseline algorithms and the simple clinical MORPH features do in terms of all six assessment metrics. Particularly, it can also be found that the SDAE algorithm can achieve a much better differentiation performance with the ALL strategy. Therefore, the involvement of more member slices as training data can be very helpful to boost the performance of a deep learning CADx scheme, even if some of the training slices only partially depict the nodules of interest. It may be because the inclusion of more nodule member slices as training data can provide richer image contexts for the SDAE model to augment the differentiation capability. For breast lesion classification, the SDAE algorithm still outperforms the two texture-based algorithms as shown in Table 2, Figs 4 and 5. It is also worth noting that the data size of lung nodules is almost three times of the breast data size.

Referring to Tables 1 and 2, the CURVE and RANK algorithms perform better at their original problem than the other conventional CADx methods. For the performance comparison on the two slice selection strategies of the lung CADx problem in the Table 1, Figs 3, 4 and 5, the involvement of all slices for the CURVE and RANK algorithms doesn’t help much. In particular, the RANK algorithm with ALL strategy performs slightly worse than the RANK model trained with SINGLE strategy. This may be because the features used in the RANK algorithm are not only ineffective for the nodule differentiation but also confusing with rich image contexts presented in various member slices of nodules for the classifier. Accordingly, it can be suggested that features for the conventional CADx framework may need to be specifically designed for each problem at hand. Thus far, there is barely general features that can be effective for all kinds of diseases and modalities.

Referring to Table 1 and relevant Figs 3 and 5, it can also be found that the nodule classification performance of the CURVE algorithm with either the ALL or SINGLE strategy is not significantly different to the performance attained by the MORPH features from experts’ drawings in ACC metrics. Therefore, it suggests that the elaboration of feature extraction for a CADx may sometimes not significantly outperform the simple morphological features commonly used in clinical practice. Accordingly, the design of effective features for a specific CADx problem can be an arduous problem. On the other hand, the SDAE-based CADx algorithm can do well on both problems with the same architecture setting and doesn’t require the explicit elaboration on feature extraction and feature relation establishment. Meanwhile, the SAE/SDAE models are equipped with a visualization mechanism of the learnt patterns encoded in the neurons at the unsupervised phase, as illustrated in Fig. 2, to facilitate the developer and even medical doctors to understand the machine learning model easier. These advantages and the natural *end-to-end* training manner of the deep learning methods can potentially benefit the inter-discipline application like CADx to enable people with engineering and clinical background work closer and come up with more effective solutions.

Although the SDAE algorithm cannot attain a performance as good as the earlier morphology-based framework^4^ on the breast dataset, it doesn’t need the image segmentation process to obtain a nodule/lesion boundary. The work^4^ requires a user to manually give the definition of a ROI and select one segmentation result from the five lesion boundary proposals generated by the segmentation algorithm. So far, there is no algorithm that can guarantee perfect automatic segmentation results for any object of interest and imaging modality. Note that image segmentation for pulmonary nodules in CT scans may sometimes include the nearby tissues, e.g., vessel and airway, into the segmentation results due to similar intensity distribution, whereas the accuracy of breast lesion segmentation in US images can be easily degraded with the presence of serious shadowing effect. The effect of combination of morphological and textural features on the conventional CADx has also been shown in the Tables 1 and 2. To avoid the user intervention on the segmentation process, the initialization and parameters of the DRLSE and GC methods are fixed. As it can be found in the Tables 1 and 2, the MORPH features from contours with the DRLSE and GC methods are not very helpful. It might be because the image segmentation results are imperfect and therefore the derived MORPH features are less reliable. Accordingly, this may reflect the conclusion of the work^4^ that the quality of image segmentation result matters for the CADx scheme based on the morphological features. For a better quality of morphological feature computing, the computed lesion/nodule boundaries usually require manual intervention, including model initialization^13^,^15^, parameter adjustment^6^,^13^,^15^, boundary refinement^6^ and selection^4^,^23^, to improve the segmentation results. Nevertheless, the user intervention will bias the computerized differentiation results because the process like boundary refinement and selection involves subjective judgment on the lesion/nodule. Accordingly, the pure objectiveness of computerized diagnosis may no longer hold. In contrast, the SDAE algorithm can circumvent this complex image segmentation step, but can still achieve an objective and satisfactory performance without any user intervention. The SDAE algorithm can potentially attain an even better performance if the data size of breast US is larger.

One of the major research lines on textural feature computation is based on the statistics of the Grey Level Co-occurrence Matrix (GLCM). To boost the differentiation performance, most promising texture-based CADx methods, e.g., the works^12^,^19^, decompose the raw image into independent components with a bank of filters, and then compute the GLCM from each component in multiple scales and orientations. With each GLCM, quite a few texture features can be further derived, e.g., 12 in the work^19^ and 14 in the work^12^. In such a framework, the intertwined combination of parameter settings on the filter bank, scale and orientation settings of the GLCM, and the number of derived features from each GLCM could lead to a large number of features. In this case, the feature selection step has to be applied^19^,^20^ to find a subset of the most useful features. Since the feature selection in itself is very tedious and needs to repeat the training process for many times, the whole CADx learning process will be rendered into a very long cycle. On the other hand, the training process of the deep architecture is relatively simple, but can achieve a better performance, see Fig. 1 for comparison.

In summary, the deep learning architecture holds the advantages of **1)** automatic discovery of object features, **2)** automatic exploration of feature hierarchy and interaction, **3)** relatively simple *end-to-end* training process, and **4)** systematic performance tuning. Although deep learning has been successfully applied to many image- and acoustic-related problems^46^, it is less explored in the context of CADx. In this study, the efficacy of a deep learning-based CADx scheme has been corroborated with extensive experiments. Comparing to the conventional CADx framework, the training procedure of deep learning is relatively simple but effective, without the need of explicit design of the problem-oriented features. To our best knowledge, this is the first deep-learning-based CADx study that demonstrates outperformance over the state-of-the-art CADx algorithms across different imaging modalities and diseases. This work may, thus, be referential for further deep learning studies on other CADx problems and the analysis of higher dimensional image data and images from heterogeneous imaging modalities for more accurate and reliable computerized diagnostic support.

## Methods

### Datasets

The methods were carried out by in “accordance” with the approved guidelines. All experimental protocols were approved by Taipei Veterans General Hospital and the Lung Image Database Consortium (LIDC)^47^,^48^. Informed consent was obtained from all subjects. The breast US images adopted here were acquired at Taipei Veterans General Hospital, Taipei, Taiwan^4^ with proper IRB approvals. Totally, 520 breast sonograms were scanned from 520 patients. The data involves 275 benign and 245 malignant lesions. All breast lesions were histopathologically proved by means of biopsy, mastectomy, etc. More details about the involved US breast lesions can be found in the work^4^. The Lung Image Database Consortium (LIDC)^47^,^48^ retrospectively collected lung CT image data from 1,010 patients (with appropriate local IRB approvals) at seven academic institutions with various CT machines in the United States. The slice thicknesses of the CT scans ranged diversely from 0.6 mm to 5 mm. 12 radiologists from 5 sites were involved in the annotation process. Nodules with diameters larger than 3 mm were further annotated with rating of malignancy by the radiologists. In this study, we randomly select 700 malignant and 700 benign nodules as the experimental dataset for data balanced. Specifically, the benign set includes the annotated nodules with scores of 1 and 2 in the “likelihood of malignancy” rating, whereas nodules scored as 4 and 5 are included in the malignant set.

### SDAE-based CADx framework

**Figure 6:**
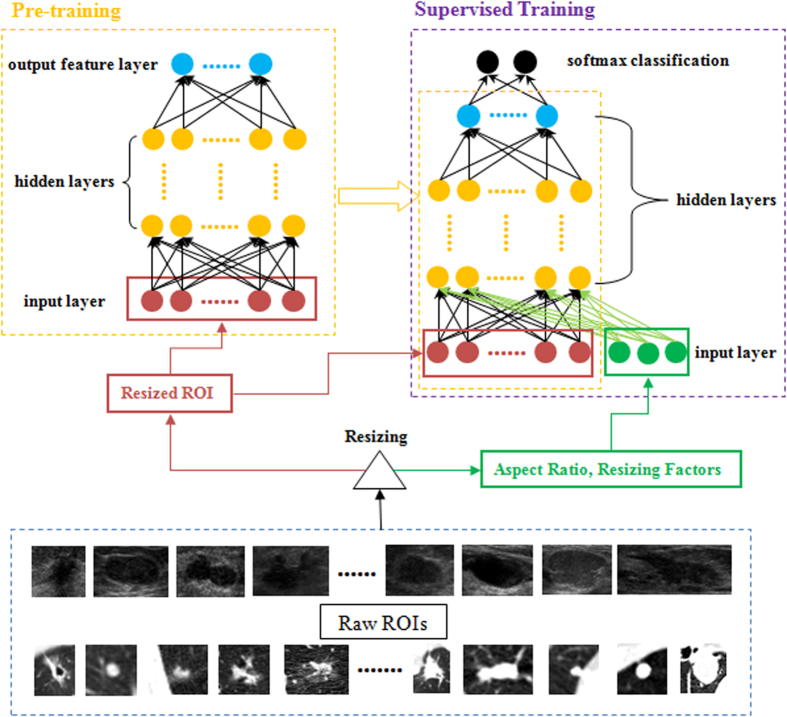
Flow-chart of our deep-learning-based CADx training framework. The pixels of resized ROIs are fed into the network architecture at the pre-training step. The pre-trained network is then refined with the supervised training by adding three neurons carrying aspect ratio of the original ROI and also the resizing factors at the input layer. The final identification result can be made with the softmax classification.

The training of a SDAE-based CADx framework can be realized in two steps: the pre-training and supervised training steps. Figure 6 illustrates the flowchart of the SDAE-based CADx framework. To facilitate the training and differentiation tasks on the SDAE architecture, the image ROIs are resized into smaller patches of 28 × 28, where all pixels in each patch are treated as the input neurons. To preserve the original information, the resized scale factors of the two ROI dimensions and the aspect ratios of the original ROIs are later added into the input layer in the supervised training step.

At the pre-training step, the input ROIs are degraded with random corruption and then the noise-tolerance representative patterns for the lesion/tumor can be further identified with the network architecture. Meanwhile, higher semantic levels of the representative patterns and the composite/interaction relations from the low semantic levels can be further sought by constructing autoencoders layer by layer, see Fig. 2. The constructed architecture at the pre-training step can be served as reliable network initialization for the latter supervised training.

The supervised training of the SDAE-based CADx framework performs the fine-tuning on the network architecture to yield the desirable discriminative performance. The scaling factors in the *x* and *y* dimensions and the aspect ratio are treated as three individual neurons as new inputs, whereas two extra output neurons of benign and malignant classes are also added on the top of the network for the supervised training, see Fig. 6. The modified network is then equipped with the initialization from the pre-trained architecture, three new input neurons, and the two outputs. Afterward, the supervised training is performed with the conventional back-propagation for the fine-tuning of the whole network. With such network architecture and two steps of training, the feature extraction and selection can be systematically and jointly realized with less need of *explicit* and *ad-hoc* elaborations. The technical explanation about the SDAE model can be found in the first section of the supplementary.

### Conventional CADx Algorithms for Comparison

Two texture-based CADx schemes for breast US^19^ and lung CT^12^ are implemented for comparison. Note that the morphological features are not considered here, since it needs an image segmentation process, which is very hard and often requires manual refinement on the automated segmentation results. On the other hand, textural features can be computed directly from the ROI. Accordingly the texture-based CADx schemes are relatively automatic and objective, and hence more suitable to serve as the comparison baselines. The implemented breast US CADx algorithm^19^, called RANK, is the state-of-the-art texture-based method for breast lesions classification. The RANK algorithm carries out the ranklet transform, which was shown to be robust to speckle noise, and to decompose the US image data into several independent image components. The GLCM-based texture features are computed from the ranklet components, whereas feature selection is conducted with the bootstrap method^19^,^20^ to find an effective subset of features for classification. We adopted the reported *best selected features*^19^ as the input to SVM to reach the final classification result for each US ROI.

For lung nodule differentiation, the latest lung CT CADx algorithm^12^, denoted as CURVE, is implemented. The CURVE algorithm applies the curvelet transform to decompose the raw image data into several sub-band components and then computes the GLCM on each component to derive texture features. To reduce feature dimensionality, the CURVE algorithm averages the textural features over all angle parameter settings of each GLCM for the later classification step with SVM. It is worth noting that the RANK and CURVE algorithms share some common steps such as image decomposition with transform techniques, computing of GLCM features, and SVM-based classification, though their feature selection and integration processes are different.

### Classification with Clinical Features

To further compare the effectiveness of the SDAE-based CADx algorithm, the simple clinical (MORPH) features, which are commonly used as preliminary reference for differential diagnosis in clinical practice, are also implemented. Specifically, the size and diameter features of the breast and nodules are computed from the US images and CT scans, respectively. For pulmonary nodules, we compute the nodule volume, the maximum major diameter of the approximate ellipsoid of the nodule contour over all member slices, and the maximum area of the nodule over all member slices as the quantitative features. It is worth noting that the computed nodule volume can only approximate the real value due to the slice thickness effect and segmentation errors. The three quantitative features are further classified with SVM for the benignancy/malignancy classification. Similarly, we also compute the simple morphological features for the US breast lesions for comparison. The specific simple morphological features are the lesion area and the length of the major axis of the approximated ellipsoid of each breast lesion. These two features are further served as input of a SVM for the breast lesion classification. All the MORPH features are derived from manual outlines from experienced medical doctors and image segmentation results from the DRLSE level set^43^ and grow-cut^44^ methods.

### Training and Testing of CADx Algorithms

Since the US breast data are 2D images, the training and testing of CADx algorithms is relatively simple. On the other hand, the image resolution of lung CT scan is anisotropic between the *z* (slice thickness) and *x*-*y* directions. The slice thickness varies widely. As the CT scan is sliced into 2D transversal views, the computation of a 3D feature on the *z* direction is less reliable than on the other *x* and *y* directions. In such a case, there will be an issue of slice selection for the representation of a nodule in the design of the CADx. For a pulmonary nodule, because in some member CT slices only small portions of the nodule are depicted, not every member CT slice of a nodule can be useful. Accordingly, the efficacy of the involvement of all CT slices for the lung CADx is unknown. Two slice selection, i.e., SINGLE and ALL, strategies are implemented in this study for the lung CT CADx problem. In SINGLE strategy, the middle slice of the nodule is selected as the representative sample. Each ROI sample stands for a distinctive nodule. In the ALL strategy, all member slices of each nodule are participating in the training and testing. In the training stage, all ROIs of the member slices from training nodules are treated as the training data. For the prediction of a testing nodule, a major voting scheme is implemented to reach the final classification result. Specifically, if more than half of the member slices are identified as malignant by the CADx model, the nodule will be regarded as a malignant nodule. In this study, the SINGLE and ALL strategies are implemented with the SDAE, CURVE, and RANK algorithms to illustrate the classification performance.

## Additional Information

**How to cite this article**: Cheng, J.-Z. *et al.* Computer-Aided Diagnosis with Deep Learning Architecture: Applications to Breast Lesions in US Images and Pulmonary Nodules in CT Scans. *Sci. Rep.* **6**, 24454; doi: 10.1038/srep24454 (2016).

## Supplementary Material

Supplementary Information

## Figures and Tables

**Table 1:** Performance summary of the SDAE, CURVE and RANK algorithms on the lung CT dataset.

LUNG		ACC (%)	AUC (%)	SENS (%)	SPEC (%)	PPV (%)	NPV (%)
SDAE1 SINGLE	**cv-all**	**87.4** ± **3.3**	**94.1** ± **1.9**	**86.3** ± **5.6**	**88.5** ± **4.8**	**86.9** ± **4.5**	**88.8** ± **4.1**
	cv1	88.6 ± 2.5	95.0 ± 2.1	88.1 ± 3.8	88.0 ± 5.5	89.2 ± 3.1	88.4 ± 4.3
	cv5	87.3 ± 3.2	93.5 ± 1.9	86.9 ± 4.3	87.7 ± 5.8	87.1 ± 3.5	89.4 ± 3.6
	cv10	86.6 ± 4.1	93.5 ± 1.7	87.4 ± 4.2	85.9 ± 7.4	87.3 ± 3.8	88.1 ± 4.8
CURVE1 SINGLE	**cv-all**	**77.8** ± **2.9**	**86.0** ± **2.6**	**75.9** ± **4.9**	**79.6** ± **3.6**	**76.9** ± **3.8**	**78.9** ± **3.1**
	cv1	78.4 ± 2.3	86.1 ± 2.9	76.6 ± 4.4	80.1 ± 3.5	77.5 ± 3.2	79.5 ± 2.8
	cv5	77.4 ± 4.6	86.0 ± 4.0	76.1 ± 8.2	78.6 ± 4.1	77.1 ± 6.6	78.0 ± 3.6
	cv10	77.2 ± 3.1	85.7 ± 2.9	75.9 ± 5.1	78.6 ± 4.4	76.6 ± 3.9	78.1 ± 3.7
RANK1 SINGLE	**cv-all**	**75.9** ± **3.1**	**81.8** ± **3.4**	**73.8** ± **4.9**	**78.0** ± **4.4**	**75.0** ± **3.6**	**77.1** ± **3.6**
	cv1	75.6 ± 3.5	81.5 ± 3.5	73.9 ± 5.8	77.4 ± 4.6	74.9 ± 4.3	76.7 ± 3.8
	cv5	75.5 ± 1.6	81.6 ± 3.7	73.1 ± 3.8	77.9 ± 3.5	74.5±2.4	76.9 ± 2.5
	Cv10	76.0 ± 3.9	82.0 ± 4.7	74.0 ± 5.3	78.0 ± 4.8	75.1 ± 4.3	77.2 ± 4.3
SDAE2 ALL	**cv-all**	**94.4** ± **3.2**	**98.4** ± **1.5**	**90.8** ± **5.3**	**98.1** ± **2.2**	**91.6** ± **4.4**	**97.9** ± **2.5**
	cv1	95.6 ± 3.0	98.9 ± 1.0	92.4 ± 5.4	98.9 ± 1.3	93.1 ± 4.6	98.8 ± 1.4
	cv5	94.6 ± 2.5	98.5 ± 1.7	90.9 ± 4.1	98.3 ± 2.3	91.6 ± 3.5	98.3 ± 2.4
	cv10	93.6 ± 3.1	98.4 ± 1.1	89.9 ± 4.2	97.3 ± 2.7	90.6 ± 3.7	97.3 ± 2.9
CURVE2 ALL	**cv-all**	**78.4** ± **2.9**	**85.1** ± **2.7**	**75.1** ± **4.5**	**81.7** ± **4.2**	**76.7** ± **3.3**	**80.5** ± **3.7**
	cv1	78.9 ± 3.1	85.0 ± 2.5	75.3 ± 6.1	82.4 ± 2.5	77.2 ± 4.3	81.1 ± 2.5
	cv5	78.4 ± 3.8	85.2 ± 3.1	74.3 ± 4.8	82.6 ± 5.3	76.3 ± 3.7	81.2 ± 5.0
	cv10	77.1 ± 1.5	84.7 ± 3.1	74.1 ± 4.1	80.1 ± 4.2	75.7 ± 2.1	79.1 ± 3.0
RANK2 ALL	**cv-all**	**72.0** ± **3.5**	**74.6** ± **4.1**	**62.0** ± **5.4**	**82.0** ± **4.2**	**68.4** ± **3.3**	**77.6** ± **4.4**
	cv1	72.5 ± 3.6	74.9 ± 5.0	63.3 ± 5.5	81.7 ± 4.2	69.1 ± 3.4	77.7 ± 4.6
	cv5	71.8 ± 2.9	74.4 ± 3.5	61.7 ± 4.4	81.9 ± 5.4	68.2 ± 2.4	77.6 ± 4.9
	Cv10	72.0 ± 2.5	74.1 ± 2.7	61.4 ± 3.6	82.6 ± 2.8	68.2 ± 2.3	77.9 ± 3.2
MORPH MAN	**cv-all**	**78.1** ± **3.3**	**86.6** ± **3.0**	**70.9** ± **5.1**	**88.5** ± **4.8**	**74.7** ± **3.4**	**83.1** ± **4.3**
	cv1	78.1 ± 4.1	86.5 ± 3.0	70.9 ± 4.2	88.0 ± 5.5	74.6 ± 3.5	83.1 ± 5.8
	cv5	78.4 ± 3.4	86.7 ± 2.8	71.0 ± 6.9	87.7 ± 5.8	75.0 ± 4.2	83.4 ± 2.4
	Cv10	78.0 ± 1.8	86.6 ± 2.3	70.7 ± 3.6	85.9 ± 7.4	74.5 ± 1.9	83.0 ± 3.8
MORPH DRLSE	**cv-all**	**71.8** ± **3.7**	**76.2** ± **3.6**	**63.3** ± **5.7**	**80.4** ± **4.4**	**68.8** ± **3.6**	**76.5** ± **4.5**
	cv1	72.1 ± 2.8	76.3 ± 2.5	63.6 ± 5.7	80.7 ± 3.8	69.0 ± 3.3	76.8 ± 3.3
	cv5	71.5 ± 5.0	76.0 ± 5.1	62.7 ± 8.4	80.3 ± 4.6	68.5 ± 5.3	76.0 ± 4.8
	Cv10	71.8 ± 3.7	76.0 ± 3.4	63.4 ± 5.0	80.1 ± 4.4	68.7 ± 3.5	76.1 ± 4.1
MORPH GC	**cv-all**	**73.3** ± **3.7**	**78.8** ± **3.3**	**66.2** ± **5.4**	**80.3** ± **4.8**	**70.5** ± **3.6**	**77.2** ± **4.5**
	cv1	73.5 ± 1.8	78.7 ± 1.7	66.7 ± 3.3	80.3 ± 3.6	70.7 ± 1.8	77.3 ± 2.8
	cv5	73.6 ± 3.5	78.9 ± 3.6	66.4 ± 7.0	80.7±3.6	70.8 ± 4.0	77.5 ± 3.3
	Cv10	73.1 ± 3.7	78.7 ± 3.3	66.0 ± 3.3	80.3 ± 3.6	70.3 ± 2.5	77.1 ± 3.7
CURVE1 DRLSE SINGLE	**cv-all**	**75.7** ± **3.7**	**83.9** ± **3.5**	**67.3** ± **6.3**	**84.0** ± **5.0**	**72.2** ± **3.9**	**81.0** ± **4.8**
	cv1	76.4 ± 3.8	85.3 ± 4.1	69.0 ± 5.3	83.9 ± 5.0	73.1 ± 3.7	81.2 ± 5.2
	cv5	75.8 ± 2.7	83.1 ± 3.2	67.3 ± 3.0	84.3 ± 3.0	72.0 ± 2.4	81.1 ± 3.4
	Cv10	74.3 ± 3.5	83.2 ± 2.9	65.9 ± 8.7	82.7 ± 3.9	71.2 ± 5.0	79.3 ± 3.1
CURVE1 GC SINGLE	**cv-all**	**76.3** ± **3.7**	**84.4** ± **3.4**	**67.0** ± **6.3**	**85.6** ± **4.8**	**72.3** ± **3.9**	**82.5** ± **4.8**
	cv1	78.0 ± 3.9	85.0 ± 4.0	69.7 ± 3.6	86.3 ± 5.4	74.0 ± 3.1	83.7 ± 5.8
	cv5	75.9 ± 2.7	83.9±3.0	66.3 ± 5.3	85.8 ± 3.1	71.9 ± 3.3	82.2 ± 3.1
	Cv10	75.4 ± 1.7	84.3 ± 2.4	65.6 ± 5.6	85.3 ± 3.6	71.4 ± 2.7	81.9 ± 2.8
RANK1 DRLSE SINGLE	**cv-all**	**75.9** ± **4.2**	**82.7** ± **4.2**	**74.9** ± **5.9**	**76.9** ± **6.7**	**75.5** ± **4.5**	**76.7** ± **5.3**
	cv1	77.5 ± 5.2	84.0 ± 4.9	76.1 ± 5.6	78.9 ± 9.0	76.8 ± 4.7	78.9 ± 9.0
	cv5	74.5 ± 3.2	81.8 ± 3.9	75.6 ± 3.8	73.4 ± 3.8	75.1 ± 3.5	73.4 ± 3.8
	Cv10	76.5 ± 3.8	82.5 ± 2.7	75.7 ± 4.8	77.3 ± 5.5	76.2 ± 4.0	77.3 ± 5.5
RANK1 GC SINGLE	**cv-all**	**75.4** ± **4.0**	**82.0** ± **4.0**	**72.1** ± **6.0**	**78.7** ± **5.7**	**74.0** ± **4.3**	**77.4** ± **4.8**
	cv1	76.2 ± 4.7	83.0 ± 5.2	71.6 ± 7.2	80.9 ± 6.2	74.2 ± 5.0	79.2 ± 6.1
	cv5	75.3 ± 4.8	81.5 ± 3.0	72.9 ± 5.1	77.7 ± 7.0	74.1 ± 4.2	76.8 ± 5.7
	Cv10	75.1 ± 4.1	81.6 ± 3.2	72.7 ± 5.9	77.6 ± 5.8	74.1 ± 4.3	76.6 ± 4.9
CURVE2 DRLSE ALL	**cv-all**	**76.6** ± **3.1**	**85.4** ± **2.8**	**68.0** ± **5.5**	**85.2** ± **4.2**	**72.8** ± **3.4**	**82.3** ± **4.1**
	cv1	77.2 ± 2.7	85.3 ± 2.6	68.6 ± 5.8	85.9 ± 2.6	73.4 ± 3.4	82.9 ± 2.5
	cv5	76.3 ± 3.9	85.3 ± 2.3	68.0 ± 5.5	84.6 ± 3.4	72.6 ± 3.9	81.5 ± 4.0
	Cv10	76.3 ± 3.8	85.5 ± 3.6	68.0 ± 5.6	84.6 ± 5.5	72.7 ± 3.8	81.8 ± 5.2
CURVE2	**cv-all**	**78.3** ± **3.5**	**85.5** ± **3.0**	**75.7** ± **5.5**	**81.0** ± **4.2**	**77.1** ± **4.2**	**80.0** ± **3.8**
GC	cv1	78.6 ± 4.6	85.4 ± 3.8	76.1 ± 4.5	81.1 ± 5.8	77.3 ± 4.2	80.3 ± 5.6
ALL	cv5	78.6 ± 4.0	85.3 ± 3.2	76.3 ± 5.9	80.9 ± 3.6	77.5 ± 4.6	79.9 ± 3.7
	Cv10	78.2 ± 4.0	85.2 ± 2.4	75.1 ± 6.5	81.3 ± 2.6	76.8 ± 4.9	80.0 ± 3.1
RANK2 DRLSE ALL	**cv-all**	**69.1** ± **3.7**	**83.1** ± **3.3**	**48.2** ± **6.4**	**90.0** ± **3.8**	**63.6** ± **3.1**	**82.9** ± **5.6**
	cv1	69.6 ± 3.3	82.9 ± 2.6	48.7 ± 5.4	90.4 ± 3.6	63.9 ± 2.6	83.7 ± 5.4
	cv5	69.1 ± 2.8	83.6 ± 2.9	48.1 ± 5.4	90.1 ± 2.8	63.5 ± ± 2.3	83.1 ± 4.1
	Cv10	69.1 ± 4.2	83.2 ± 3.1	48.1 ± 7.5	90.0 ± 3.9	63.6 ± 3.7	82.9 ± 5.6
RANK2 GC ALL	**cv-all**	**72.2** ± **3.3**	**83.8** ± **3.1**	**57.8** ± **5.8**	**86.5** ± **4.1**	**67.3** ± **3.0**	**81.3** ± **4.6**
	cv1	73.0 ± 2.2	83.9 ± 2.4	58.6 ± 4.2	87.4 ± 3.3	67.9 ± 2.2	82.5 ± 3.6
	cv5	72.5 ± 2.6	83.8 ± 1.7	58.1±5.7	86.9 ± 3.4	67.6 ± 2.7	81.7 ± 3.3
	Cv10	71.6 ± 2.6	84.1 ± 3.1	57.3 ± 6.2	86.0 ± 2.5	67.0 ± 2.8	80.4 ± 2.4
CURVE1 MAN SINGLE	**cv-all**	**77.0** ± **3.6**	**85.9** ± **2.9**	**69.3** ± **5.8**	**84.7** ± **4.0**	**73.5** ± **3.9**	**82.0** ± **4.2**
	cv1	76.6 ± 4.8	85.6 ± 3.4	69.3 ± 6.5	84.3 ± 3.0	73.4 ± 4.8	81.4 ± 5.7
	cv5	76.6 ± 3.7	85.6 ± 2.7	68.9 ± 6.2	84.0 ± 5.4	73.2 ± 3.0	81.4 ± 3.4
	Cv10	76.4 ± 3.2	85.6 ± 2.6	68.1 ± 3.8	84.7 ± 3.6	72.7 ± 2.9	81.7 ± 4.1
RANK1 MAN SINGLE	**cv-all**	**74.9** ± **3.3**	**83.9** ± **3.0**	**66.6** ± **5.5**	**83.2** ± **4.5**	**71.5** ± **3.4**	**80.0** ± **4.2**
	cv1	75.7 ± 3.4	83.9 ± 2.0	68.0 ± 7.6	83.4 ± 3.0	72.6 ± 4.7	80.4 ± 2.7
	cv5	75.1 ± 4.3	83.9±3.3	66.9 ± 5.2	83.3 ± 5.4	71.6 ± 4.0	80.2 ± 5.5
	Cv10	74.4 ± 2.1	84.0 ± 2.7	66.3 ± 5.3	82.6 ± 3.6	71.1 ± 2.8	79.3 ± 2.9
CURVE2 MAN ALL	**cv-all**	**77.0** ± **3.6**	**85.9** ± **2.9**	**69.3** ± **5.8**	**84.5** ± **4.0**	**73.5** ± **3.9**	**82.0** ± **4.2**
	cv1	76.6 ± 4.8	85.6 ± 3.4	69.3 ± 6.5	84.0 ± 5.4	73.4 ± 4.8	81.4 ± 5.7
	cv5	76.6 ± 3.7	85.6 ± 2.7	68.9 ± 6.2	84.3 ± 3.0	73.2 ± 4.0	81.4 ± 3.4
	Cv10	76.4 ± 3.2	85.6 ± 2.6	68.1 ± 3.8	84.7 ± 3.6	72.7 ± 2.9	81.7 ± 4.1
RANK2 MAN ALL	**cv-all**	**72.9** ± **3.8**	**83.5** ± **3.1**	**59.4** ± **6.0**	**86.7** ± **4.1**	**68.1** ± **3.2**	**81.7** ± **4.7**
	cv1	73.4 ± 4.0	83.6 ± 3.0	60.0 ± 6.4	87.0 ± 4.7	68.5 ± 3.8	82.3 ± 5.3
	cv5	73.2 ± 3.4	84.1 ± 2.5	59.0 ± 6.5	87.4 ± 3.0	68.2 ± 3.4	82.5 ± 3.6
	Cv10	73.0 ± 3.3	83.4 ± 2.9	59.4 ± 6.0	86.6 ± 4.1	68.2 ± 3.2	81.7 ± 4.7

The classification performances with simple size and diameter features are also summarized in the rows denoted as MORPH. The notations “MAN”, “DRLSE”, and “GC” suggest the usage of experts’ drawings and image segmentation results from the DRLSE and GC methods, respectively, in the experiments. The rows “SDAE1”, “CURVE1”, and “RANK1” report the performance statistics of using SINGLE strategy for each algorithm, whereas the performances statistics in the rows of “SDAE2”, “CURVE2”, and “RANK2” are the results with ALL strategy in the training of each algorithm, “AUC”, “ACC”, “SENS”, “SPEC”, “PPV”, and “NPV” represents six assessment metrics: area under receiver operating characteristic curve, accuracy, sensitivity, specificity, positive predictive value, and negative predictive value, respectively. The rows “cv-all” represents the performance of each algorithm over all 100 folds, whereas the rows “cv1”, “cv5”, and “cv10” list the first, fifth, and tenth cross validations sorted by the “ACC” values of the SDAE algorithm.

**Table 2:** Performance summary of the SDAE, CURVE and RANK algorithms along with the clinical MORPH features on the breast US dataset.

BREAST		ACC (%)	AUC (%)	SENS (%)	SPEC (%)	PPV (%)	NPV (%)
SDAE	**cv-all**	**82.4** ± **4.5**	**89.6** ± **3.9**	**78.7** ± **8.0**	**85.7** ± **6.8**	**82.2** ± **5.4**	**83.4** ± **6.6**
	cv1	83.4 ± 4.6	90.0 ± 4.3	80.0 ± 5.2	87.0 ± 7.9	83.1 ± 3.9	85.1 ± 8.4
	cv5	82.5 ± 4.5	90.6 ± 3.8	78.4 ± 10.6	86.2 ± 6.9	82.4 ± 7.1	84.1 ± 6.1
	cv10	81.7 ± 6.0	89.6 ± 6.4	78.3 ± 7.1	84.7 ± 9.9	81.6 ± 5.0	83.0 ± 9.3
CURVE	**cv-all**	**75.9** ± **6.0**	**81.5** ± **5.9**	**75.6** ± **8.0**	**76.4** ± **8.3**	**78.4** ± **6.5**	**73.9** ± **5.9**
	cv1	75.6 ± 4.2	80.3 ± 5.8	76.8 ± 3.7	74.3 ± 6.6	73.9 ± 4.4	77.2 ± 4.9
	cv5	77.5 ± 7.0	82.4 ± 6.9	76.7 ± 10.4	78.4 ± 9.4	75.8 ± 8.6	80.2 ± 7.3
	cv10	74.0 ± 9.5	80.1 ± 9.0	71.9 ± 12.6	76.3 ± 9.7	71.5 ± 10.2	77.2 ± 8.7
RANK	**cv-all**	**77.9** ± **5.4**	**85.8** ± **4.7**	**76.3** ± **7.6**	**79.6** ± **7.4**	**75.3** ± **6.4**	**80.1** ± **6.1**
	cv1	77.7 ± 8.8	85.7 ± 6.6	77.6 ± 11.5	77.9 ± 7.6	76.1 ± 10.6	79.6 ± 7.2
	cv5	77.5 ± 4.4	85.6 ± 4.9	76.0 ± 6.5	79.2 ± 6.8	74.9 ± 5.3	80.6 ± 5.4
	cv10	76.7 ± 8.0	85.1 ± 7.3	77.1 ± 8.6	78.3 ± 11.0	75.4 ± 8.3	80.4 ± 8.8
MORPH MAN	**cv-all**	**72.4** ± **5.5**	**77.2** ± **6.2**	**67.6** ± **8.3**	**77.9** ± **7.8**	**68.5** ± **6.0**	**77.7** ± **6.5**
	cv1	72.3 ± 4.6	77.1 ± 6.6	67.3 ± 7.7	78.0 ± 7.2	68.3 ± 5.7	77.8 ± 6.0
	cv5	72.1 ± 6.9	77.4 ± 7.2	67.6 ± 8.7	77.1 ± 7.5	68.2 ± 7.0	76.9 ± 7.2
	cv10	72.7 ± 8.3	77.1 ± 5.6	67.3 ± 14.4	78.8 ± 7.4	68.1 ± 6.6	77.9 ± 7.0
MORPH DRLSE	**cv-all**	**64.5** ± **5.4**	**76.1** ± **8.2**	**35.0** ± **10.5**	**90.8** ± **6.1**	**61.3** ± **3.9**	**77.8** ± **13.1**
	cv1	65.5 ± 4.0	76.1 ± 11.6	37.9 ± 10.2	90.2 ± 4.8	62.2 ± 3.0	77.9 ± 7.3
	cv5	64.6 ± 5.9	76.4 ± 9.3	35.1 ± 9.7	90.9 ± 6.9	61.1 ± 4.1	78.7 ± 14.8
	Cv10	63.7 ± 3.3	76.3 ± 7.0	32.7 ± 10.5	91.3 ± 4.5	60.5 ± 2.6	78.0 ± 13.1
MORPH GC	**cv-all**	**69.5** ± **5.8**	**73.0** ± **6.4**	**63.3** ± **8.7**	**75.1** ± **9.6**	**69.8** ± **5.5**	**70.2** ± **8.5**
	cv1	71.0 ± 6.0	73.0 ± 8.2	64.6 ± 9.5	76.8 ± 8.7	71.1 ± 5.7	71.7 ± 8.0
	cv5	69.4 ± 5.9	72.0 ± 6.1	64.9 ± 9.7	73.5 ± 9.7	70.3 ± 5.4	69.0 ± 7.4
	Cv10	68.7 ± 5.9	72.5 ± 6.4	62.4 ± 8.5	74.2 ± 11.7	70.0 ± 4.3	69.6 ± 10.0
CURVE DRLSE	**cv-all**	**73.4** ± **6.4**	**78.7** ± **7.0**	**72.5** ± **9.0**	**74.3** ± **8.6**	**71.9** ± **7.7**	**75.5** ± **6.7**
	cv1	75.2 ± 7.5	80.6 ± 6.6	75.1 ± 9.0	75.4 ± 10.6	73.6 ± 9.3	77.5 ± 6.7
	cv5	73.8 ± 7.3	79.3 ± 8.5	74.3 ± 7.7	73.4 ± 9.8	71.9 ± 8.5	76.2 ± 6.7
	Cv10	71.2 ± 7.9	77.4 ± 7.5	71.8 ± 13.0	70.5 ± 7.0	68.3 ± 7.2	74.6 ± 10.1
CURVE GC	**cv-all**	**75.0** ± **5.7**	**81.0** ± **6.3**	**75.6** ± **8.3**	**74.4** ± **8.5**	**72.9** ± **6.9**	**77.7** ± **6.5**
	cv1	76.3 ± 6.5	82.7 ± 8.4	76.4 ± 8.4	76.3 ± 6.8	74.3 ± 6.8	78.5 ± 7.0
	cv5	75.2 ± 6.9	80.1 ± 6.9	72.7 ± 10.4	77.5 ± 9.5	74.8 ± 7.9	76.6 ± 7.1
	Cv10	73.8 ± 4.2	79.8 ± 5.0	77.6 ± 6.1	70.6 ± 5.4	70.2 ± 4.7	78.1 ± 4.9
RANK DRLSE	**cv-all**	**76.8** ± **5.0**	**85.4** ± **4.3**	**75.1** ± **9.0**	**78.7** ± **8.7**	**74.4** ± **6.6**	**80.4** ± **6.3**
	cv1	78.8 ± 7.0	85.5 ± 6.7	74.9±10.0	83.3 ± 8.4	75.2 ± 7.9	83.7 ± 7.8
	cv5	76.3 ± 3.0	85.4 ± 3.6	74.6 ± 7.5	78.4 ± 6.9	73.8 ± 5.4	79.8 ± 4.7
	Cv10	76.0 ± 5.2	84.6 ± 4.6	73.8 ± 13.9	78.4 ± 8.2	74.3 ± 9.5	74.3 ± 9.5
RANK GC	**cv-all**	**77.3** ± **4.2**	**85.9** ± **4.1**	**74.8** ± **7.7**	**80.1** ± **7.7**	**74.3** ± **5.5**	**81.3** ± **5.8**
	cv1	78.1 ± 3.4	85.7 ± 3.6	77.1 ± 4.9	79.1 ± 4.3	75.6 ± 4.1	80.7 ± 3.2
	cv5	77.3 ± 4.3	85.9 ± 3.4	73.1 ± 7.2	82.0 ± 9.5	73.3 ± 4.3	82.8 ± 3.2
	Cv10	76.3 ± 3.3	86.3 ± 3.5	75.3 ± 8.0	77.6 ± 4.3	74.2 ± 6.0	79.1 ± 2.7
CURVE MAN	**cv-all**	**74.0** ± **5.8**	**80.1** ± **6.6**	**73.0** ± **8.7**	**74.9** ± **7.7**	**72.5** ± **7.1**	**76.0** ± **6.7**
	cv1	75.4 ± 6.4	79.0 ± 7.4	74.3 ± 13.2	76.5 ± 12.0	74.7 ± 9.0	78.4 ± 10.2
	cv5	74.4 ± 6.2	79.7 ± 5.4	73.9 ± 6.9	74.9 ± 7.1	72.6 ± 7.1	74.3 ± 7.5
	Cv10	72.5 ± 7.4	77.6 ± 8.3	71.0 ± 9.3	73.9 ± 11.0	71.6 ± 10.4	78.1 ± 4.9
RANK MAN	**cv-all**	**77.9** ± **5.1**	**86.6** ± **4.5**	**75.0** ± **8.9**	**81.2** ± **7.5**	**74.9** ± **6.7**	**82.1** ± **6.1**
	cv1	78.7 ± 6.0	86.8 ± 3.6	72.8 ± 9.6	85.4 ± 8.4	74.2 ± 7.2	85.1 ± 7.3
	cv5	78.1 ± 4.0	85.7 ± 3.3	74.6 ± 7.9	82.1 ± 5.0	74.6 ± 5.9	82.5 ± 4.1
	Cv10	76.7 ± 6.5	86.7 ± 5.2	73.9 ± 14.3	80.1 ± 8.9	74.6 ± 9.7	81.3 ± 6.7

The notations “MAN”, “DRLSE”, and “GC” suggest the usage of experts’ drawings and image segmentation results from the DRLSE and GC methods, respectively, in the experiments. “AUC”, “ACC”, “SENS”, “SPEC”, “PPV”, and “NPV” represents six assessment metrics: area under receiver operating characteristic curve, accuracy, sensitivity, specificity, positive predictive value, and negative predictive value, respectively. The rows “cv-all” represents the performance of each algorithm over all 100 folds, whereas the rows “cv1”, “cv5”, and “cv10” list the first, fifth, and tenth cross validations sorted by the ACC values of the SDAE algorithm.
