# Supplementary material for: Computer-Aided Diagnosis with Deep Learning Architecture: Applications to Breast Lesions in US Images and Pulmonary Nodules in CT Scans
Source: Sci Rep. 2016 Apr 15;6:24454. doi: 10.1038/srep24454 (PMC4832199; doi:10.1038/srep24454)
Supplement: Supplementary Information [file srep24454-s1.pdf]

Computer-Aided Diagnosis with Deep Learning Architecture: Applications to Breast Lesions in US Images and Pulmonary Nodules in CT Scans

Jie-Zhi Cheng[1], Dong Ni[1], Yi-Hong Chou[3*], Jing Qin[1], Chui-Mei Tiu[3], Yeun-Chung Chang[4], Chiun-Sheng Huang[5], Dinggang Shen[6*], Chung-Ming Chen[2*]

[1]National-Regional Key Technology Engineering Laboratory for Medical Ultrasound, School of Medicine, Shenzhen University, Shenzhen, Guangdong 518060, P.R. China

[2]Institute of Biomedical Engineering, National Taiwan University, Taipei 100, Taiwan

[3]Department of Radiology, Taipei Veterans General Hospital and National Yang Ming University, Taipei 112, Taiwan

[4]Department of Radiology and Medical Imaging, College of Medicine, National Taiwan University, Taipei 100, Taiwan

[5]Department of Surgery, College of Medicine, National Taiwan University, Taipei 100, Taiwan

[6]Department of Radiology and Biomedical Research Imaging Center, The University of North Carolina at Chapel Hill, Chapel Hill, NC 27599, USA

*dinggang_shen@med.unc.edu, chung@ntu.edu.tw, yhchou@vghtpe.gov.tw

1. Technical explanation of SDAE method

The stacked autoencoder (SDAE) architecture can be constructed by the basic building block of autoencoder. An autoencoder is a two layered neural network that attempts to seek the representative patterns (mostly sparse), denoted as s, of the original data d, at the hidden (second) layer with the deterministic encoder mapping:

$$s = f_\theta(d) = \sigma(Wd + p) \tag{S1}$$

, where $\theta = \{W, p\}$ is the parameter set of synaptic weightings and bias, respectively, and the mapping σ is usually specified as the nonlinear sigmoid function. The hidden representations are sought by minimizing the difference between the original data d and the reconstructed data z from the hidden layer s, which can be defined as:

$$z = f'_\vartheta(s) = \sigma(W's + q) \tag{S2}$$

, where $\vartheta = \{W', q\}$ is the parameter set of synaptic weightings and bias for the reconstruction mapping,

respectively. With the building block of autoencoder, a higher level of semantic representations for the object class can be further constructed by stacking up autoencoders into deeper layers.

To improve the robustness of the SAE architecture against the noise and cluttered background, Vincent *et al.*[42] proposed to randomly corrupt the data in the training process as the SDAE architecture. In such framework, the desirable reconstructed data $\tilde{z}$ are sought with the minimization of

$$\arg min_{W,W',p,q} L(d, \sigma(W' \sigma(W\tilde{d} + p) + q)) \quad \text{(S3)}$$

, where $L(\cdot,\cdot)$ is the lost function, and $\tilde{x}$ is the corrupted data d drawn from a stochastic mapping $q_D(\tilde{d}|d)$.

More technical details of the SDAE can be found in the work[42].

2. Discussion of image segmentation results

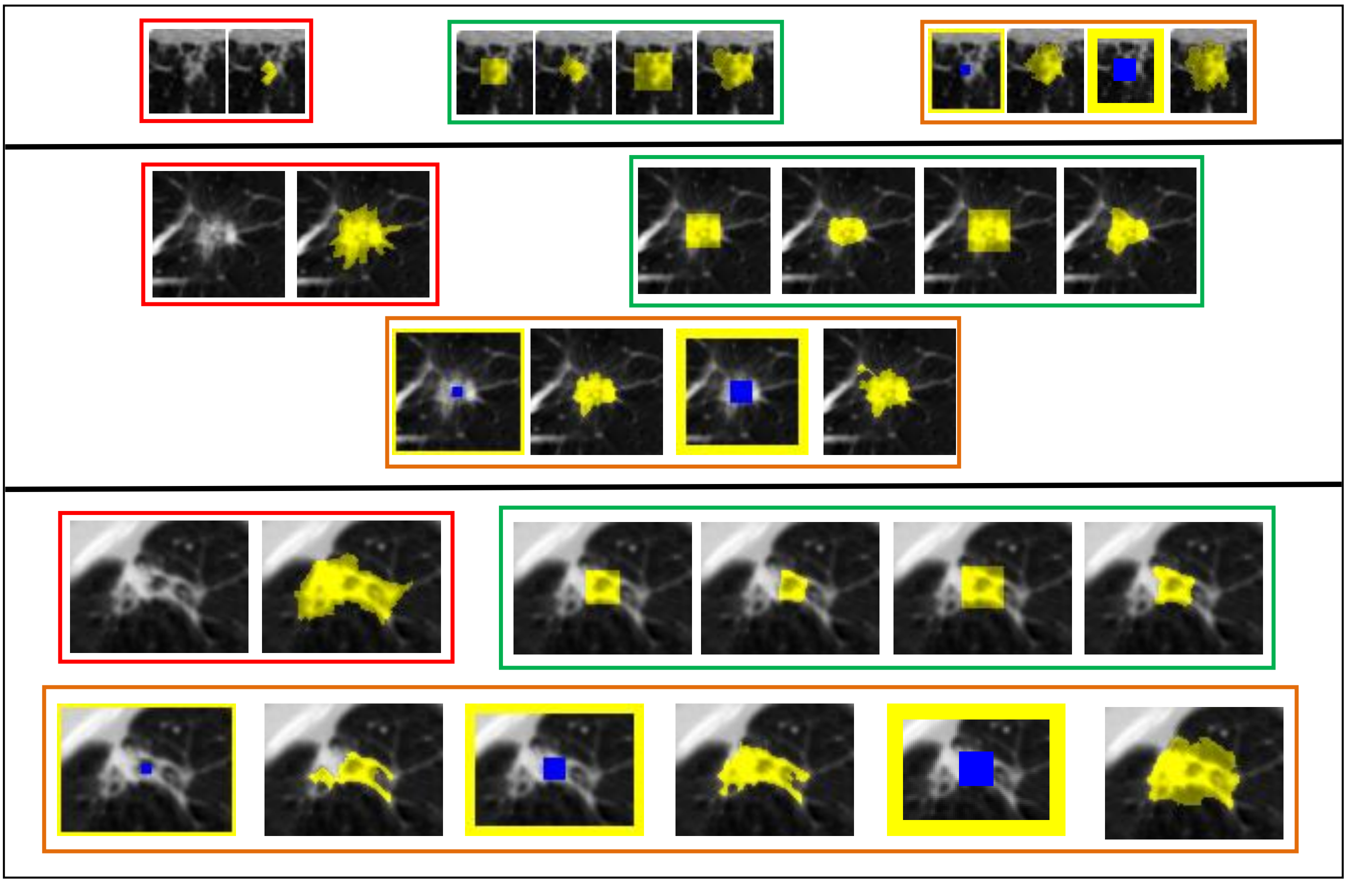

Figure S.1. Demonstration of segmentation results from the methods of DRLSE and GC on the pulmonary nodules depicted in CT scans. Three nodules with different sizes, shapes, and intensity patterns are shown in this figure with the separation of dark bold lines. For each case, the original images and the manual drawings, DRLSE results and GC results are identified with red, green and orange rectangles, respectively. In the red rectangles, the original image and the corresponding manual drawings (marked with yellow color) are lists in the left and right, respectively. The effect of different initialization for the three segmentation methods are shown in the green and orange rectangles. For the DRLSE method, the yellow boxes are the initialization of the level sets, followed by the results shown on the right hand side. The blue boxes and yellow rectangles for the GC method stand for the foreground and background initialization, respectively. Similarly, the GC results are listed on the right hand side of each initialization setting. All segmentation results are marked with yellow color in this figure.

Figure S.1, Figure S.2 and Figure S.3 list several segmentation results from the DRLSE level set, and

grow-cut (GC) methods to illustrate the effect of various settings of initialization and iteration number on the final segmentations. It can be found that one parameter setting can't be applied to all cases to achieve satisfactory segmentation results, since the size, shape, and intensity patterns of the nodule/lesion vary significantly. Therefore, to provide effective morphological features, the process of parameter tuning by the user deems to be necessary. In our experience, the parameter tuning for the level set methods is relatively difficult as the level set models have many parameters, e.g., number iterations, time steps, weightings of different terms, etc. For GC method, the major parameter lies in the setting of initialization seeds. Meanwhile, since the image properties of CT scan and sonography are different, the parameter settings of the DRLSE level set method are different in our experiments for the CADx performance comparison.

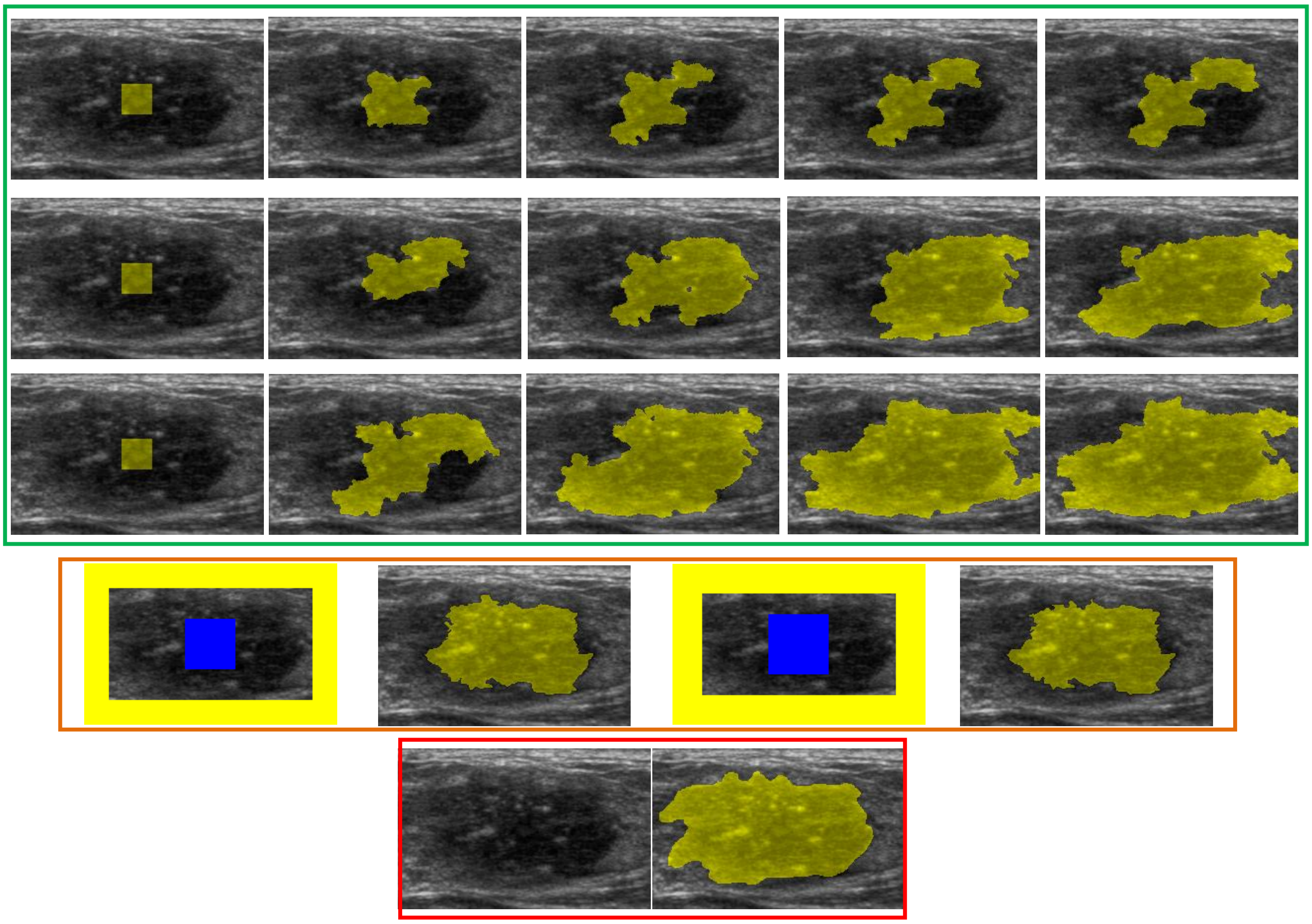

Figure S.2. Segmentation demonstration for a case of breast lesion in US image. Similar to the Fig. S.1, the red, green and orange rectangles enclose the original and manual drawings, DRLSE, and GC segmentation results, respectively. All segmentation results, including the manual outline, are marked with yellow color. For the DRLSE level set, the initializations are yellow boxes, whereas the blue and yellow regions are the initialization of foreground and background for the GC method, respectively. For the DRLSE level set method (green rectangle), we list the intermediate curve evolutions at different iteration numbers (150, 300, 450, 500 in the second, third and fourth columns, respectively) with respect to the setting of time step parameter (100, 200, 400 as the first, second and third rows). For some cases, it requires larger setting of time step while for some other cases smaller time step is preferable. The GC segmentation results are relatively stable for this case.

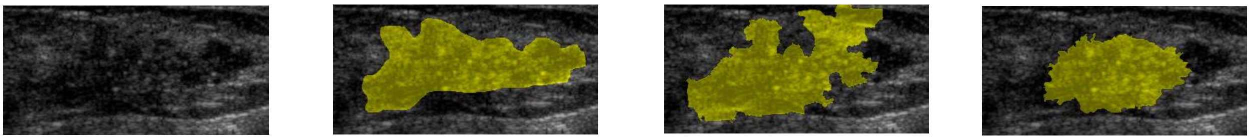

Figure S.3. Segmentation demonstration for a difficult case of US breast lesion. From the left to right, the first one is the original image and the second one is the manual outline from a medical doctor. The third and fourth ones are the results of DRLSE level

set and GC methods, respectively. The segmentation results shown in this figure are the best results by the three segmentation methods. Particularly, for the DRLSE level set, it needs to set the time step parameter as 1000 to achieve such segmentation result. Smaller setting of time step cannot drive the level to cover most parts of this breast lesion. All segmentations are marked by yellow color. It can be found that the segmentation results by the three methods are not very close to the manual outline from a senior medical doctor.
